# Distinct cholinergic circuits underlie discrete effects of reward on attention

**DOI:** 10.3389/fnmol.2024.1429316

**Published:** 2024-08-29

**Authors:** Kelly Runyon, Tung Bui, Sarah Mazanek, Alec Hartle, Katie Marschalko, William Matthew Howe

**Affiliations:** School of Neuroscience at Virginia Tech, Blacksburg, VA, United States

**Keywords:** acetylcholine, attention, reward, striatum, midbrain, dopamine, frontal cortex

## Abstract

Attention and reward are functions that are critical for the control of behavior, and massive multi-region neural systems have evolved to support the discrete computations associated with each. Previous research has also identified that attention and reward interact, though our understanding of the neural mechanisms that mediate this interplay is incomplete. Here, we review the basic neuroanatomy of attention, reward, and cholinergic systems. We then examine specific contexts in which attention and reward computations interact. Building on this work, we propose two discrete neural circuits whereby acetylcholine, released from cell groups located in different parts of the brain, mediates the impact of stimulus-reward associations as well as motivation on attentional control. We conclude by examining these circuits as a potential shared loci of dysfunction across diseases states associated with deficits in attention and reward.

## Introduction

Our brains are faced with an enormously difficult task; to steer us through a dynamic environment imbued with possibility. To guide behavior effectively, brains have evolved circuitries to determine what information is allowed to dominate ongoing cognitive processing (attention) as well as the attractiveness and value of internal states and external stimuli (reward). Functionally, attention and reward are inextricably linked, and while many studies have explored the brain circuits that underlie attention and reward independently, relatively less is known regarding the systems that stand at the interface between these functions. Given their capacity to modulate activity across wide expanses of brain circuits known to mediate attention and reward processing, ascending neuromodulator systems (e.g., those responsible for producing the neurotransmitters dopamine, serotonin, norepinephrine, and acetylcholine) are uniquely situated to mediate this interaction. Here, we bring together anatomical, physiological, and behavioral evidence to propose that central cholinergic systems are particularly important for controlling these interactions between attention and reward. We first review evidence concerning the circuit-level organization of brain reward and attention systems, and current hypotheses of physiological and behavioral functions specifically controlled by forebrain cholinergic signaling. We then build upon previous work to propose a model whereby cholinergic systems mediate the influence of central representations of stimulus-reward associations and motivation on attentional control. Given that many neuropsychiatric and neurodegenerative disorders are associated with impairments in attention and reward processing, we conclude by exploring this perspective in the context of the pathophysiology of behavioral and cognitive impairments common to both neuropsychiatric and neurodegenerative diseases.

## Attention: functions and neuroanatomy

The construct of attention has long been a focus of both psychological and neuroscientific research. The pioneering psychologist William James famously said “Everyone knows what attention is. It is the taking possession by the mind, in clear and vivid form, of one out of what seem several simultaneously possible objects or trains of thought. Focalization, concentration, of consciousness are of its essence. It implies withdrawal from some things to deal effectively with others and is a condition which has a real opposite in the confused, dazed, scatterbrained state…” (James, 1890). As described by James, ‘attention’ is fundamentally a blanket term, used to apply to several complimentary operations that collectively organize perception and behavior. Early work by Broadbent (1954) envisioned attention as a filter; a collection of cognitive processes that enabled an individual to select certain stimuli from the environment and grant privileged access to associative learning and memory systems. Work by Posner and colleagues (Posner and Boies, 1971) created a more formalized framework for deployment of attention which included maintenance of general alertness and anticipatory monitoring of an environment for the appearance of instructive stimuli over time, as well as stimulus detection, which in Posner’s terms was an explicitly cognitive process that selects a stimulus from the environment to guide behavior. This conceptualization of attention assumes that its efficacy is bounded by the central processing capacity of a given individual (i.e., multitasking; Posner and Boies, 1971), and thus individual variation therein could seed both perceptual and cognitive abnormalities. Further refinements of this theory more formally operationalized the three main components of attention described above; alertness, orienting, and stimulus detection (Posner and Petersen, 1990). Alertness refers to a global and fundamentally neurobiological state that facilitates the relay of an input to primary sensory cortices and the decoding of its physical properties. Orienting refers to the process of either covertly or overtly biasing sensory processing to the predicted source of an instructive stimulus. Detection is the vehicle for conscious awareness of that stimulus and includes activation of learned associations regarding the predictive value of that stimulus that in turn guide behavior. Theories of attention have also long attempted to reconcile the multiple ways in which the construct can be activated or “recruited.” Voluntary or “top-down” attention refers to situations in which an individual’s goals and previous experience are used to guide attentional selection. In contrast, reflexive or “bottom-up” refers to scenarios in which the properties of a sensory stimulus (e.g., brightness, volume, and surprise) capture attentional resources (Posner et al., 1980; Jonides and Irwin, 1981; Desimone and Duncan, 1995; Corbetta and Shulman, 2002). This top-down/bottom-up framework has been incredibly influential and seeded the discovery of dedicated brain circuits that are differentially engaged in these contexts (Corbetta and Shulman, 2002). However, more recent work has highlighted situations in which this dichotomy falls apart, particularly in contexts where reward-associated stimuli are presented in tandem (Awh et al., 2012). Finally, a key component of all theories of attention is the capacity to focus on instructive stimuli while willfully ignoring task-irrelevant (distracting) sensory inputs (e.g., reading this manuscript while someone nearby is having a conversation), commonly referred to as “distracter filtering”. Current models suggest that both stimulus properties and cognitive demand can influence susceptibility to distraction. Specifically, when presented with complex visual and auditory stimuli subjects are less susceptible to the influence of distracting stimuli, but more susceptible when engaged in tasks with high cognitive demand (Lavie et al., 2004; Lavie, 2005, 2010).

Given the multi-faceted nature of attention, it is perhaps unsurprising that research aimed at determining the neurobiological mechanisms that underlie it have identified a sprawling system encompassing structures from the brainstem to the frontal lobe. While the complete neuroanatomy of attention-related brain areas is outside the scope of this review, it should be noted that the parietal lobe has been shown to be critical for directing attention and enabling shifts of attention between stimulus locations and modalities (Yin and Mountcastle, 1977, Posner and Petersen, 1990). Indeed, patients with lesions in the parietal lobes exhibit a phenomenon referred to as spatial neglect and are seemingly incapable of focusing on stimuli within the receptive field of the affected hemisphere (Brain, 1941). Popular theories based on data from humans and primate (e.g., Corbetta and Shulman, 2002) as well as rodents (Bucci et al., 1998) suggest that such parietal areas are particularly important for bottom-up or surprise-induced changes in attention, perhaps due in part to their connectivity with limbic structures like the dorsal posterior cingulate cortex, which has been implicated in orienting toward a stimulus (Vogt et al., 2006). Areas of the prefrontal cortex (PFC), in contrast, are typically described as important for ‘top-down’ or goal directed attention (Nagahama et al., 2001; Corbetta and Shulman, 2002; Buschman and Miller, 2007; Li et al., 2010), although it is important to note that in the literature a number of functionally and anatomically distinct subregions are often labeled as “PFC”, making it difficult to get a clear picture of the precise functions supported by discrete areas within the frontal lobe. A major barrier to higher granularity in the causal role of different subregions of the PFC to attentional control stems from anatomical differences between humans and the model systems typically used to test the necessity and sufficiency of brain areas for different functions. Inconsistencies in PFC nomenclature applied to model systems research further complicates efforts to integrate data across species. These issues have led to several recent revisions of rodent PFC anatomy and terminology (e.g., Laubach et al., 2018). For the purposes of this review, we note that in primates the term PFC is often used to refer to granular cortex that is largely absent in rodents (Preuss and Wise, 2022), whereas anterior cingulate (ACC) is a term typically used in reference to agranular subregions of the primate frontal lobe (Laubach et al., 2018). Within the rodent literature, studies commonly refer to the prelimbic (PrL) and infralimbic (IL) subregions of the medial frontal wall as “PFC” or “mPFC,” and more dorsal and posterior frontal cortical regions are referred to as cingulate cortex (e.g., Cg1/2). Further confounding the matter, some authors refer to the whole of the rodent frontal cortex as the anterior cingulate cortex (ACC), in reference to the largely agranular composition of the rodent frontal lobe (Vogt and Gabriel, 1993; Vogt et al., 2013). Recently researchers have made efforts towards establishing a more useful cross-species terminology that focuses on the major subdivisions of the rodent PFC and their potential homologies with the primate cortex (e.g., Laubach et al., 2018). In this schema, rodent PrL is roughly equivalent to pregenual ACC in the primate, IL with subgenual ACC, and the more dorsal and posterior cingulate cortex (e.g., Cg1 and Cg2) as synonymous with anterior midcingulate cortex (aMCC; Fillinger et al., 2017; Laubach et al., 2018; van Heukelum et al., 2020). Importantly, all these frontal cortex subregions have all been linked to the control of attention in primates and rodents. For example, studies in rodents have shown that lesions encompassing the PrL and IL selectively impair stimulus-guided attentional performance and not working memory (Kahn et al., 2012), and suggest these structures might be particularly critical when tasks require the ability to shift attention between perceptual domains (Birrell and Brown, 2000). The rodent aMCC in contrast, appears necessary for the acquisition of stimulus-response contingencies in attention tasks (Bussey et al., 1997), and interestingly, may act in concert with the PrL to maintain attentional focus in anticipation of instructive task cues (Totah et al., 2013). Research from primates and humans also suggests the posterior MCC may be key for orienting toward the location of a stimulus and shifting of attention based on cognitive demand, arousal, and awareness (Kulkarni et al., 2005; Leech and Sharp, 2014; Vogt, 2016). Together, studies from rodents, non-human primates, and humans all highlight the importance of parietal and frontal lobe structures for the control of different aspects of attention, though clearly more work is needed to understand the unique computations supported by the subregions within each.

Outside of the cortex, multiple thalamic nuclei appear to be important for attentional control. The pulvinar nuclei, which share reciprocal connections with the superior colliculus, visual cortex, and provide input to multiple frontal cortical areas, have been suggested to be key for both attentional orienting and the filtering of distracting stimuli (Posner and Petersen, 1990; Fischer and Whitney, 2012). In addition, projections from the medio-dorsal thalamus have been suggested to be key for relaying stimulus information to the cortex so that associative information imparted by a predictive stimulus may be used to guide behavior (Parikh and Sarter, 2008; Parikh et al., 2010; Hasselmo and Sarter, 2011; Saalmann and Kastner, 2015). Notably, the thalamic reticular complex or nucleus, a band of GABAergic cells that encapsulates the thalamus, has also been proposed to serve as an “attentional searchlight,” effectively focusing such thalamic input to the cortex via targeted inhibition (Crick, 1984; McAlonan et al., 2000; Pinault, 2004).

## Reward: functions and neuroanatomy

The term ‘reward’ is one that is commonly used by both the scientific and non-scientific community. Its commonality has created a certain amount of historical confusion around the way the term is being operationalized in different contexts. Like attention, in the scientific literature ‘reward’ is shorthand for several related functions that serve to gate the probability of behaviors proximal to the presentation of a stimulus. Berridge and Robinson (2003) proposed that reward can be broken into 3 separable, but connected, functional domains which we will focus upon here: affect/hedonics, reinforcement/associative learning, and motivation. The domain of hedonics chiefly concerns the affective experience of pleasure (positive) or disgust (negative). These hedonic signals are typically associated with the subjective experience of a primary reward like food or sex (Berridge and Kringelbach, 2011). These subjective effects can be enormous, such as the pleasure associated with the taste of a favorite food when hungry, or conversely visceral malaise following food poisoning (Cabanac, 1992; Rolls, 2005). Certain drugs of abuse, in particular opiates, are also capable of generating intense subjective experiences of pleasure (Doyle et al., 1993; Berridge et al., 2009). In humans, such hedonic encoding can also be applied to more abstract rewards, like art or social interactions (Berridge and Kringelbach, 2011; Goller et al., 2019), highlighting the interplay between learning and affective representations. In addition to interacting with affect, associative learning processes comprise their own unique domain of reward. Reinforcement learning refers to a particular class of associative learning focused on optimizing the outcomes of behavior and guided by representations of the relationship between predictive stimuli, actions, and value of a primary reward. Importantly, such learning is distinct from the hedonic impact (e.g., pleasure or disgust) associated with the unconditioned stimulus (or “reward”), though the two information streams likely interact. These learned and reinforced associations are often described as either model-free or model-based. The most straightforward way of conceptualizing the distinction between these two models is to first consider a primary reward (food or sex) as an unconditioned stimulus (something with innate value), and a neutral cue that predicts its future availability. In model-free learning, a learned association is an inevitable consequence of repeated pairing of the neutral cue with subsequent reward. While the association between cue and reward is shaped by the magnitude or “value” of the unconditioned stimulus, a conscious representation of a reward’s value, or the pleasure associated with receiving it, is not necessary to establish this relationship. In contrast, model-based learning assumes there is an internal, necessarily neurobiological, representation of need, probability, or magnitude of the predicted reward that influences the strength of the association. A key feature of such model-based associations is that these internal representations of reward value or contingency can be engaged to economize the decision-making process (Kahneman, 2003; Daw et al., 2005, 2011; Dayan and Berridge, 2014). Importantly, both forms of learning are accepted as playing major roles in guiding reward-related behavior, and while historically considered as separable functions likely controlled by dissociable brain networks, more recent work suggests that the two work in concert via overlapping circuitry (e.g., O’Doherty et al., 2017). The third domain of reward concerns the construct of motivation. Motivation is typically used to refer to a process or force that controls the vigor of ongoing behavior. It has alternatively been labeled as the desire, or “wanting”, for a particular reward or reward-paired stimulus (Berridge et al., 1989; Robinson and Berridge, 1993). Theories of motivation as a construct can be traced back to the work of Clark Hull, who posited that internal states drive an agent to satisfy physiological needs like access to food, water, and mates (Hull, 1943). Later work expanded upon these ideas to more fully incorporate the capacity of cues that have been paired with primary rewards to become potent activators of appetitive behaviors. Such ‘incentive stimuli’ take on reward-like qualities and can serve as reinforcers in and of themselves (Bindra, 1969; Bolles, 1972; Toates, 1986; Robinson and Berridge, 1993). Modern theories of motivation place varying amounts of emphasis on these two sources of drive, though each shares the capacity to trigger, attract, and guide appetitive behavior.

Like attention, the ability of behavior and cognition to be shaped by reward represents a major adaptive advantage, and our brains have evolved brain circuits to facilitate their encoding and representation (Nesse, 2002; Berridge and Kringelbach, 2008). The first set of experiments looking into the idea of localized reward centers was conducted in 1954 by Olds and Milner (1954), and demonstrated that stimulating the medial forebrain bundle, which is comprised of fibers connecting the hindbrain, midbrain, and forebrain, produced a range of reinforcement behaviors (Olds and Milner, 1954). In the years since this pioneering work, our understanding of how each of the domains of reward function are represented in the brain has become more in-depth, implicating a vast network of cortical and subcortical structures. Interestingly, while different studies have highlighted support for a variety of subcircuits in different domains of reward function, it is clear certain structures are key for each; the striatum (namely dorsal medial striatum (DS) and nucleus accumbens (NAc) core and shell, the orbitofrontal cortex (OFC), and the amygdala (basolateral and centro-medial). Manipulation of activity in the striatum, particularly the shell of the NAc, is key for modifying the experience of sensory pleasure (Castro and Berridge, 2014a). Work in rodents, primates, and humans has also demonstrated strong support for the OFC as a hub for neural encoding of pleasure associated with primary tastes (Castro and Berridge, 2017), smell (de Araujo et al., 2003), and sex (Buchel et al., 2018). The amygdala, which receives input from ascending and descending gustatory systems, provides strong input to the shell of the nucleus accumbens, and has long been suggested to represent a region where sensory inputs are ascribed with hedonic reward qualities (Aggleton, 1986; Zahm, 1999). Though unlike the striatum and OFC, evidence is less clear, and several studies have noted a lack of modification of sensory pleasure following amygdala manipulations (e.g., Castro and Berridge, 2014a). These same regions have also been identified by studies probing the neurobiological underpinnings of associative reward learning. OFC lesions reduce the capacity for rats to discriminate between good and bad predictors of reward (Ostlund and Balleine, 2007). The OFC is essential for encoding food-motivated associative learning (Aou et al., 1983; Ross et al., 2005), and shifting actions following a change in outcome value (Gremel et al., 2016), supporting a role for the OFC in specifically model-based learning (Huang et al., 2020). Similarly, lesions of the dorsal medial striatum (DMS) (Yin et al., 2005), NAc core and shell (Noguer-Calabús et al., 2022), and amygdala (Málková et al., 1997; Balleine et al., 2003) all impair the ability of animals to update their behavior based on changes in reward value and contingency. The amygdala shares connections with the OFC and is a major source of excitatory input to the striatum, and manipulations of OFC to basolateral amygdala (BLA) connectivity can differentially modulate value updating and retrieval, highlighting the importance of inter-connectivity within this circuitry (Malvaez et al., 2019). It is also worth noting that the importance of the OFC, amygdala, and striatum in both the encoding and retrieval of reward values (e.g., Balleine et al., 2003; Yin et al., 2005; Ostlund and Balleine, 2007; Malvaez et al., 2019; Lichtenberg et al., 2021; Sias et al., 2021) highlights the dynamic interplay between reward learning and memory systems that future studies should interrogate more explicitly. Striatal and amygdala circuitries have also been shown to be critical for both global motivational drive as well as the attribution of motivational value to reward paired cues (e.g., Holland and Gallagher, 2003; Corbit and Balleine, 2005; Talmi et al., 2008; Winstanley et al., 2010; Saunders et al., 2013, 2018). The contribution of OFC to motivation, independent of behavioral modifications stemming from updated representations of reward value, is less clear, though again multiple studies have noted correlations between OFC function and motivated behavior (Gallagher et al., 1999; Arana et al., 2003; Cetin et al., 2004).

An important question that emerges from this work highlighting shared neural circuitry across reward domains is how discrete functions are encoded within them. One possible explanation is that different domains of reward are encoded by unique patterns of neuromodulation that shift the activity state of local microcircuitries within these structures. Harkening back to the original experiments by Olds and Milner, midbrain dopamine (DA) systems are known to modulate multiple reward functions and as such, are a key part of the larger neuroanatomy of reward. Midbrain DA systems can be roughly segregated into two separate projection systems. The nigro-striatal system begins in the substantia nigra pars compacta (SNc) and largely projects to the DS as well as the central amygdala (Poulin et al., 2018), pallidum (Lavoie et al., 1989), subthalamic nucleus (Cragg et al., 2004), and motor thalamus (Antal et al., 2014). The meso-cortico-limbic pathway stems from the ventral tegmental area (VTA) and is the primary source of DA innervation of the NAc, frontal association cortex, BLA, and other components of brain limbic systems (Beier et al., 2015). Early work on DA modulation of food reward led to the hypothesis that these systems in the brain encode sensory pleasure associated with a food reward (Wise et al., 1978). Direct tests of this hypothesis suggested that DA is neither necessary nor sufficient for the experience of sensory pleasure (Berridge et al., 1989; Berridge and Valenstein, 1991). Pioneering work by Montague and colleagues provided the first accounts of a shift in DA neuron activity from the receipt of an unexpected reward to the time of a cue that predicts a reward, as a mechanism of associative learning (Montague et al., 1996; Schultz et al., 1997). This demonstration seeded the hypothesis that midbrain DA systems encode a reward prediction error, or the difference in value between received and expected rewards (Montague and Berns, 2002; Schultz, 2016; Lerner et al., 2021). More recent studies have suggested that SNc and VTA DA populations uniquely contribute to different aspects of reward learning, perhaps via changes in the patterns and timescales of on-going release (Hamid et al., 2016, 2021; Saunders et al., 2018; Mohebi et al., 2019), though overlapping and distinct functions of each are still debated. Hypotheses regarding the importance of DA for reward learning are supported by historical and recent studies (e.g., Amo et al., 2022). That said, there is also a substantial body of work suggesting that DA systems are keenly involved in motivated behaviors as well. Hyper-dopaminergic mice show increased willingness to work for food rewards, supporting a role in general appetitive drive (Pecina et al., 2003). More specifically, meso-limbic DA projections to the NAc are necessary for a predictive stimulus to become a driver of behavior, suggesting that VTA to NAc DA systems are particularly important for the attribution of incentive value to reward cues (e.g., Cannon and Palmiter, 2003; Robinson et al., 2006; Palmiter, 2008; Flagel et al., 2011; Saunders and Robinson, 2012; Saunders et al., 2013; Gallardo et al., 2014). Importantly, work in rodents demonstrates that PrL, IL, and amygdala receive inputs from, and project back to, these mesolimbic reward circuitries, setting the stage for dynamic feedback loops that are engaged to support reward-based decision making (Carr and Sesack, 2000; Geisler and Wise, 2008; Jo et al., 2013; Steinberg et al., 2020). This combined work importantly highlights a key role for neuromodulation in the coding of reward, and as we move forward, we will see that like DA, ascending cholinergic systems also likely contribute to specific reward states via circuit-defined patterns of activity.

## Central cholinergic systems

Acetylcholine (ACh) was discovered in a series of experiments by Loewi (1921), providing the first demonstration of chemical signaling in the nervous system. ACh is ubiquitous in the peripheral and central nervous systems. When released, ACh exerts its effects via actions on two major receptor subtypes, muscarinic (mAChR) and nicotinic (nAChR) receptors. There are five different isoforms of mAChRs which are broadly separated into two groups: M1-like (M1, M3, and M5) which are generally post-synaptic and excitatory, and M2-like (M2, M4) which can be pre- or post-synaptic and are typically inhibitory (Kruse et al., 2014). nAChRs, in contrast, are ligand-gated cation channels that depolarize cells when opened. These pentameric receptors are composed of combinations of α- (α2–10) and β (β2–4)-subunits, the most common configurations found in the brain being the α4β2 and α7 subtypes (Leonard and Bertrand, 2001; Perry et al., 2002; Gotti and Clementi, 2004). In the brain, ACh is produced and released by a small number of neurons located in the brainstem, basal forebrain (BF), and striatum. The brainstem cholinergic system comprises two separate nuclei in the pons: the pedunculopontine nucleus (PPN) and the lateral dorsal tegmentum (LDT). These cells are important for regulating general behavioral sleep and arousal, their major projection target being the thalamus. The PPN/LDT is interconnected with another cholinergic nuclei, the basal forebrain (BF; Mesulam et al., 1983; Satoh and Fibiger, 1986; Woolf and Butcher, 1986, 1991), which in turn sends projections back to the PPN/LDT (Jourdain, 1988). Brainstem cholinergic systems also target midbrain DA cell populations of the VTA and SNc (Oakman et al., 1995; Forster and Blaha, 2000; Xiao et al., 2016), suggesting these cells may contribute to several reward and motor-related functions. Cholinergic neurons of the BF are the predominant source of acetylcholine in the cortex in rodents (Johnston et al., 1979; Wenk et al., 1980), non-human primates (Kitt et al., 1987), and humans (Mesulam and Van Hoesen, 1976; Mesulam, 1996). These cells are also the major source of ACh in the hippocampus and amygdala (Divac, 1975; Lehmann et al., 1980; Mesulam et al., 1983; Halliwell, 1989; Woolf and Butcher, 1991). The nucleus basalis of Mynert (nBM), a band of neurons within the BF, receives input from many monoaminergic systems including DA from the VTA (Fallon and Moore, 1978; Beckstead et al., 1979), serotonin from the dorsal raphe nuclei (Jones and Cuello, 1989), and norepinephrine from the locus coeruleus (Zaborszky, 1989). The BF also shares reciprocal connections with multiple areas of associative cortex important for attention and reward processing, notably the PrL and IL cortices in rodents and the OFC and temporal lobe in primates (Mesulam and Mufson, 1984; Gaykema et al., 1991; Zaborszky et al., 1997). Given this pattern of connectivity, along with the demonstration of profuse loss in the number and density of nBM neurons in brain samples from Alzheimer’s disease patients (Whitehouse et al., 1982), the BF has long been a target for research into brain systems underlying cognitive functions (Whishaw et al., 1985; Jacob Huff et al., 1988; Mandel et al., 1989). At the level of the striatum, the major source of ACh is a small population of local cholinergic interneurons (CINs; Bolam, 1984; Calabresi et al., 2000). The striatum has among the highest levels of markers for cholinergic signaling in the brain though it receives no input from the BF, and only a minor input from the PPN/LDT (Macintosh, 1941; Hebb and Silver, 1961; Woolf et al., 1984; Dautan et al., 2016). The striatum is largely a GABAergic nucleus; 95% of its neurons are GABAergic medium spiny (MSN) projection cells, and another 4% are local GABAergic interneurons. The remaining 1–2% of cells are CINs which are believed to produce a majority of striatal ACh (Woolf and Butcher, 1981; Oldenburg and Ding, 2011). First identified in 1896 by Kölliker (1896), these extensively arborized cells can directly modulate the activity state of striatal microcircuitries via muscarinic acetylcholine receptors expressed by both D1 and D2 populations of MSNs (Yan et al., 2001; Surmeier et al., 2007). CINs have large dendritic trees and because of this branching structure, they can integrate synaptic inputs over a large region (Wilson et al., 1990). Given their location in the striatum, it is perhaps not surprising that existing evidence suggests these cells may be key for modulating motor output and reward function (Pisani et al., 2007; Gritton et al., 2019; Mohebi et al., 2023). There is also a sparse population of interneurons in the cortex that are positive for choline acetyltransferase, an enzyme critical for the synthesis of ACh. These cells have been suggested to represent an additional source of cholinergic modulation in the cortex, though it should be noted that their expression varies between model organisms, and there is mixed evidence regarding their presence in humans (Hedreen et al., 1983; Mesulam et al., 1983; Avendaño et al., 1996; Raghanti et al., 2008). Research in rodent models suggests this cell population may independently contribute to attentional function, in addition to input from the basal forebrain (e.g., Obermayer et al., 2019). These interneurons are enriched in cortical layers 2/3, have a bipolar morphology, and receive excitatory and inhibitory input from nearby pyramidal cells. Their activation has an excitatory effect on surrounding cells, mediated in large part by nAChRs (e.g., Von Engelhardt et al., 2007; Obermayer et al., 2017, 2019).

## ACh in attention

Some of the first experiments into the functions of ACh in the brain provided strong evidence that cholinergic signaling can potently modulate the impact of sensory inputs on activity in primary sensory cortex (Metherate and Weinberger, 1990; Metherate and Ashe, 1991). Indeed, ACh appears to enhance the representation of thalamic input across multiple sensory cortical subregions, while simultaneously suppressing the impact of intracortical input on local activity (Hasselmo and Bower, 1992; Hasselmo and Cekic, 1996). These observations supported the general hypothesis that cholinergic inputs determine the strength of the representation of an external stimulus in sensory cortex (Donoghue, 1987; Ma et al., 1989; Tremblay et al., 1990a,b), perhaps through the modulation of local synaptic plasticity (Cole and Nicoll, 1984; Krnjević, 1993). Following the development of toxins used to selectively lesion cholinergic cells in animal models, evidence began to accumulate that suggested cortical ACh may play a more specific role in the modulation of attention. Specifically, lesions of basal forebrain via ACh-immunotoxin 192 IgG-saporin impaired performance on a sustained attention task designed for rodents, selectively reducing the capacity of rats to report the presence of instructive cues, while having no effect on their ability to report the absence of these cues (McGaughy et al., 1996). Similarly, selective cholinergic deafferentation of the entire cortical mantle was shown to impact the performance of attention tasks where animals must flexibly switch between stimulus modalities to guide behavioral selection (Turchi and Sarter, 1997). Additional work utilizing microdialysis in rats demonstrated that extra-synaptic ACh concentrations measured with a probe covering both the aMCC and parts of posterior parietal cortex greatly increase during tasks that explicitly tax attention compared to control tasks that include similar motor output and reward delivery (Arnold et al., 2002). In conjunction with primate studies demonstrating the necessity of ACh signaling for attentional modulation of neural activity in the visual cortex (Fries et al., 2001; Herrero et al., 2008), by the late 2000s the consensus in the field was that the BF-cortical cholinergic system is a chief component of brain attention networks. Further insight into the way cortical ACh contributes to attention came on the heels of the development of biosensors capable of monitoring sub-second ACh release dynamics is discrete parts of the cortex. Utilizing this technology, fast increases in ACh release were observed in the rodent PrL, but not the motor cortex, when animals successfully detected reward-predictive cues and used them to guide their subsequent behavior (Parikh et al., 2007). These data provided the first-ever demonstration of fast (e.g., seconds long) and cortex-area specific patterns of ACh release in task-performing animals, and suggested that regional patterns of ACh release are dynamic and key to the control of attention and related functions Further, such cue-evoked ACh release events develop only after animals learn the predictive value of cues, are not present when animals ignore these cues, and not observed when animals are aske to similiary indicate the absence of predictive cues (Parikh and Sarter, 2008; Howe et al., 2013). Follow up studies in rats and transgenic mice demonstrated that cue-triggered ACh release in PrL can amplify glutamate released by thalamic inputs (Parikh and Sarter, 2008; Parikh et al., 2008), increase synchrony in gamma-frequency oscillations in local cell populations via binding at both nAChRs and mAChRs (Bailey et al., 2010; Guillem et al., 2011; Howe et al., 2017; Záborszky et al., 2018; Lu et al., 2020; Yang et al., 2021), and is ultimately causal in the detection process (Gritton et al., 2016). Collapsing across data accumulated over the last four decades, it seems that BF cholinergic systems, particularly their projections to areas like the rodent PrL, are a central feature of the neuroanatomy of attention, and contribute by generating rapid bursts of release that bias activity in local cell populations such that predictive cues can gain control of ongoing decision-making machinery and guide behavior (Hasselmo and Sarter, 2011). Whether this effect of ACh on post-synaptic cells populations is ‘transient’ or can additionally influence attentional performance by supporting local synaptic remodeling as demonstrated in sensory cortex, is currently unknown, though it is interesting to speculate that ACh might be operating over multiple timescales to support both on-going cue-based decision making and facilitating future attentional performance by re-wiring cortical micro-circuitries.

## ACh in reward

Each of the major brain cholinergic systems have been linked to reward-related computations as well. Selective recordings of calcium dynamics in brainstem cholinergic neurons of mice have demonstrated robust modulation of neuronal activity around the time of receiving a food reward (Ruan et al., 2022). Similarly, studies in humans have shown that PPN activity is modulated by reward receipt, and further, electrical stimulation of the PPN selectively enhances reward (but not punishment)-based learning (Skvortsova et al., 2021). Interestingly, single unit recordings from presumptive cholinergic cells in primate PPN identified neurons that were modulated by reward expectation and others that were responsive reward receipt, suggestive of distinctly tuned cholinergic populations in the brainstem (Kobayashi and Okada, 2007). Physiological correlates of reward-related computations have been described in CINs of the striatum as well. In Pavlovian cue-reward paradigms, CINs respond to a predictive stimulus only when it is followed by reward (Apicella et al., 1991; Graybiel et al., 1994). CINs specifically exhibit a pause in firing following salient stimuli (Aosaki et al., 1994), which may be linked to the encoding of reward presentation (Shimo and Hikosaka, 2001) and facilitate information provided by coincident DA signals (Cragg, 2006). Selective inhibition of CINs in the NAc core increases, and optogenetic stimulation reduces, reward-seeking behavior (Collins et al., 2019). Conversely, blockade of muscarinic receptors in the NAc shell with non-selective antagonist scopolamine reduces both the pleasure associated with a food reward, as well as the motivation to seek it (Perry et al., 2009; Castro et al., 2016). This disparity could indicate region-specific encoding of reward by striatal CINs, though it should be noted that local scopolamine application has been shown to increase ACh release (Parikh et al., 2004). Striatal ACh has also been linked to the maintenance of flexibility in reward-guided behavior; the disruption of which could contribute to habitual reward-seeking phenotypes that have been associated disorders like drug abuse (Ragozzino, 2003; Ragozzino et al., 2009; Bradfield et al., 2013; Matamales et al., 2016; Favier et al., 2020). Neurophysiological evidence also suggests that BF cholinergic neurons signal reward and punishment through changes in their spiking activity as well. Single unit recordings with the BF revealed that populations of cholinergic neurons respond to both appetitive rewards and aversive stimuli, and moreover, the magnitude of these responses scale with predicted outcome values, similar to the activity of DA neurons in cued-reward tasks (Hangya et al., 2015). ACh from BF terminals is released in the BLA in response to reward predictive cues, and such release facilitates cue-reward learning (Crouse et al., 2020). Conversely, BLA ACh release, in interaction with local norepinephrine signaling, also appears to contribute to the modulation of behaviors associated with negative affective states (Mineur et al., 2016, 2018, 2022; Sizer et al., 2022).

The literature linking the activity of ACh systems to the control of specific reward behaviors is growing. Notably, brain cholinergic systems are anatomically well positioned to directly modulate the activity of midbrain DA systems, and thereby the multiple reward functions linked to DA release. DA neurons express both mAChRs and nAChRs, and nAChR modulation of DA is believed to be a key feature in pathological reward seeking in addiction (de Kloet et al., 2015; Grasing, 2016). As stated above, output from brainstem cholinergic systems directly targets DA neurons in the VTA and SNc (Dautan et al., 2014; Mena-Segovia and Bolam, 2017). Input from brainstem cholinergic neurons to SNc DA neurons can cause these cells to burst-fire, suggesting they can directly support the capacity of phasic DA to encode reward (Hong and Hikosaka, 2014). A similar result has been reported at the level of brainstem inputs to the VTA (Xiao et al., 2016), and further, activation of LDT-NAc pathway independently increases motivation, induces place preference, and drives positive reinforcement (Coimbra et al., 2019). Regarding CINs, their stimulation in the nucleus accumbens can directly drive DA release (Cachope et al., 2012). Interestingly, reward-related pauses in CIN activity are dependent upon DA receptor binding on CINs, suggesting that reward functions are likely dependent on a delicate balance of ACh and DA release in the striatum (Zhang et al., 2018; Gallo et al., 2022; Mohebi et al., 2023). Unlike brainstem and striatal cholinergic systems, evidence for direct control of VTA DA neurons by BF cholinergic neurons is lacking, though interestingly both GABAergic and glutamatergic projection neurons of the BF do target the midbrain, modulate DA neuron activity, and subsequent reward-related behaviors (Cai, 2020; Wang et al., 2021). Furthermore, the VTA sends projections back to the BF (Swanson, 1982; Gaykema and Zaborszky, 1996, 1997; Hu et al., 2016; Gielow and Zaborszky, 2017), again highlighting the structural and functional intermingling of these two systems.

## Cholinergic control of interactions between attention and reward

A robust literature supports the general notion that internal representations of reward influence our decision of what to pay attention to. For example, the ability of a stimulus to capture attention is determined by the magnitude of reward it has been paired with, or its validity as a predictor of reward (Poh et al., 2019; Kaskan et al., 2022). High value rewards are particularly potent attractors of attention, and stimuli associated with them can become a source of distraction if presented in the context of other goal directed actions (Anderson et al., 2011; Watson et al., 2020). For example, Anderson et al. (2011) trained human participants to associate a color with a monetary reward. Subjects then completed a separate visual search task, and even though contextually irrelevant, presentation of that color increased the amount of time it took subjects to identify the task-relevant stimulus. Similarly, Watson et al. (2020) showed that there is a relationship between reward magnitude and time it takes one to avert their attention away from the stimulus that produced that reward previously. Together, studies from human subjects support the notion that stored representations of reward recruited by such cues can modulate the deployment of attention across multiple contexts (Anderson et al., 2011; Watson et al., 2020). Evidence from animal models also supports this relationship and provides insights into the neural mechanisms that mediate interactions between attention and reward. When trained on a Pavlovian cue-reward association where the extension of lever into an operant chamber predicts a food reward, rodents develop a conditioned response to lever extension, though this response varies between subjects. Some animals begin to approach the reward-predictive lever when it is extended into the chamber, interacting with it as though it was a food item (Sign trackers, ST). Such ST rats appear to place greater incentive value to reward-paired cues, are more sensitive to the effects of drug-paired cues on drug seeking behavior, and even show differences in the profile of DA release in the NAc and PrL in response to reward cues (Saunders and Robinson, 2010; Flagel et al., 2011; Meyer et al., 2012; Sarter and Phillips, 2018). Other animals instead approach the location where food rewards will eventually be presented (Goal trackers, GT) (Meyer et al., 2012). Follow-up studies in ST and GT rats revealed that ST are uniquely impaired in attentional control, have lower levels of attention-task-related acetylcholine release, show alterations in the cellular tracking of the high-affinity choline transporter that ultimately reduce their capacity to sustain PrL ACh release, and are more responsive to the sensory characteristics of salient external stimuli (Paolone et al., 2010; Cherian et al., 2017; Phillips and Sarter, 2020). These rodent studies provide a compelling demonstration that the cognitive and neural systems that control reward evaluation do not occur in a vacuum, but necessarily impact attentional function and associated brain systems (e.g., forebrain cholinergic).

Above we addressed the processes that contribute to attention and reward, described their associated neuroanatomy, and presented evidence suggesting that cholinergic systems support each through actions within these brain circuits. Next, we will specifically examine evidence regarding neurobiological scaffolds that enable interactions between attention and reward. We conclude by proposing separable circuitries linking the frontal cortex with mesolimbic systems whereby cholinergic systems act to mediate the interactions between attention, representations of reward value, and motivation.

## Cholinergic mechanisms mediating the influence of reward value on attention

**FIGURE 1:**
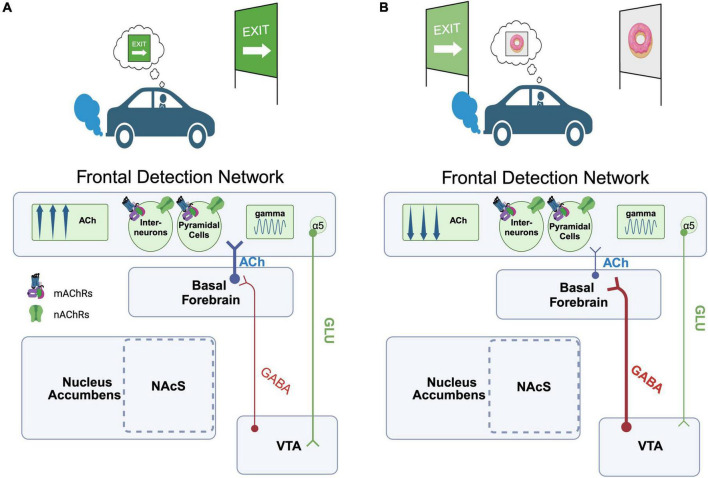
Proposed circuitry underlying the capacity of value representations to modulate cue-detection during attention-task performance. **(A)** Scenario 1. An individual is driving down the road monitoring for a road sign (cue) that indicates their intended exit. Detection of the road sign cue evokes an increase in ACh within a frontal cue detection network (or “FDN,” encompassing rodent PrL or functionally homologous structures in primates), synchronizing gamma oscillations that in turn enable the road sign cue to be used to guide behavior. FDN projection cells in layers 5/6 enriched in the α5 nAChR in turn project downstream to the VTA, which may shape activity in midbrain ensembles encoding this cue-reward association. **(B)** Scenario 2. A second cue, previously associated with a high value reward (a donut), is unexpectedly encountered. Midbrain GABAergic projections to the BF are recruited, reducing FDN ACh release and interfering with the ability of the individual to detect the exit sign cue relevant for their current goal. Created with BioRender.com.

Our primary hypothesis is that representations of the value of primary rewards and reward-paired cues are a major source of bias for the online control of attention, and this process depends on both dopamine and acetylcholine release (Hasselmo, 2006; Huang and Li, 2022). However, it is important to note that early learning of cue-reward associations is also likely to be dependent upon dopamine and acetylcholine release. We propose that each of these attention-reward interactions is differentially dependent upon ACh release from cholinergic cell groups in the basal forebrain and brainstem, respectively. Beginning with early cue-reward learning, brainstem cholinergic neurons of the PPN provide direct input to midbrain DA cells, which are enriched in nAChRs (de Kloet et al., 2015). Electrophysiological recordings of PPN neurons during reward conditioning tasks reveals that separable populations of PPN neurons respond phasically to auditory and visual stimuli (tones and rewards; Pan and Hyland, 2005). Importantly, these responses are present prior to these cues being paired with rewards and precede the DA neuron responses that these cues evoke after pairing. Thus, PPN input to the VTA may relay sensory modality-specific “bottom-up” information that alerts or primes DA neurons to the presence of a sensory stimulus, and thereby facilitate the establishment of a cue-reward association (Pan and Hyland, 2005). While this PPN input may facilitate the early stages of DA encoding of reward, the activity profile of these cells doesn’t appear to change dramatically across learning (Pan and Hyland, 2005). It therefore seems unlikely that they are a major substrate of the brain circuits that allow learned cue-reward associations to modulate attention. For this interaction, we draw attention to excitatory inputs to the dopaminergic midbrain from the frontal cortex, including the PrL and IL cortices of rats (Carr and Sesack, 2000; Geisler and Wise, 2008) and ACC and OFC of primates (Frankle et al., 2006). The PrL area in rodents, and its functional homologues in the primate cortex, have demonstrated to be key for the control of behaviors guided by representations of reward value, expectation, and error (Corbit and Balleine, 2003; Amiez et al., 2005; Rudebeck and Murray, 2014; Alexander and Brown, 2019). This region shares connections with nearly every node within the reward system, as well as other frontal cortical areas including aMCC, OFC, and IL (Carr and Sesack, 2000; Briand et al., 2007; Geisler and Wise, 2008; Wallis and Kennerley, 2010; Jo et al., 2013; Ferenczi et al., 2016). Indeed, previous rodent studies have also demonstrated that PrL input to the VTA can potently modulate the activity of local GABAergic neurons that in turn control DA neuron excitability (Jo et al., 2013), affording the PrL with the capacity to gate DA release and associated reward computations. As described above, these same frontal cortical regions (e.g., PrL/IL in rats) are critical components of the brain systems that enable attentional control and are reciprocally connected with the basal forebrain (Gaykema et al., 1991; Bloem et al., 2014). This combined pattern of connectivity suggests that these circuits connecting areas like PrL to ascending DA and ACh projection systems are uniquely situated to serve as a nexus of attention and reward. Building off these findings, we propose a circuit model whereby mesolimbic reward computations can directly impact cue-detection and attentional control. To conceptualize the functional interaction supported by this circuitry, we imagine an individual on a long car trip, constantly monitoring the roadside for the sign that indicates the exit they need to take to ultimately arrive at their desired destination. In the first scenario (Figure 1A), the individual successfully detects the exit sign. This detection event is supported by a transient increase in ACh release evoked in frontal cue detection networks (‘FDN’) as described above (e.g., PrL of rodents, analogous structures in primates; Parikh et al., 2007; Howe et al., 2013). This cue-evoked increase in ACh synchronizes oscillatory activity within the gamma frequency band through activation of local nAChRs and mAChRs, located on local interneurons and pyramidal cells within the FDN (Poorthuis et al., 2013; de Kloet et al., 2015; Howe et al., 2017). The induction of this high-frequency synchrony by ACh in turn modifies the strength of output from the FDN to the VTA. Importantly, VTA-projecting FDN neurons of deep layers 5/6 are enriched in the α5 nAChR subunit, which increases the responsivity of pyramidal cells to nAChR stimulation (Bailey et al., 2010; Poorthuis et al., 2013; Howe et al., 2018). Thus, detection-evoked ACh release in the FDN may recruit a descending pathway to the midbrain that allows attentional networks to shape the activity of DA projection neurons, and the cue-reward associations they encode. In an alternative scenario (Figure 1B), we imagine that while the individual is driving and scanning for the exit sign, they suddenly encounter an advertisement from a purveyor of their favorite treat: hot, delicious donuts. Drawn in by the promise of this potent primary reward signaled by the advertisement, the individual misses the sign that indicates their intended exit. Functionally, the presentation of the donut sign, a conditioned stimulus associated with an alternative source of reward, decreased the ability of the task-relevant cue (the exit sign) to capture their attention. Mechanistically, this interference could stem from direct interactions between the VTA/SNc and basal forebrain. Neurons of the VTA/SNc send efferent projections to the basal forebrain (Zaborszky and Cullinan, 1996; Gaykema and Zaborszky, 1997). This projection appears to be largely GABAergic (Gaykema and Zaborszky, 1997), though modern tracing studies suggest the possible existence of a minor projection from Th+ cells in the VTA to the BF (Gielow and Zaborszky, 2017). GABAergic neurons of the VTA are particularly responsive to reward cues (Wakabayashi et al., 2019), and gate value signals generated by DA cells (Eshel et al., 2015). We suggest that the unexpected presentation of this secondary reward cue (donut sign) activates a subset of midbrain GABA neurons that then project to cholinergic cells of the basal forebrain. Activation of this pathway could then hyperpolarize FDN-projecting BF cholinergic neurons, thereby reducing both the ACh release evoked by the exit sign (Gritton et al., 2016; Howe et al., 2017) and the probability that it can capture attention and guide subsequent behavior.

Although the above discussion largely focuses on how learned cue-reward associations may interfere with ongoing attentional performance, it’s important to note that the brain most likely evolved circuitry to allow for such reward-based interference because it serves an adaptive advantage. Rewards are attributed with high value when they satisfy some biological or cognitive need, and thus an attention system that is oblivious to cues that signal the sudden availability of high value rewards could impair fitness. We propose that the circuitry described above represents an evolutionarily conserved mechanism for disengaging attentional focus from a current goal and enabling the subject to re-orient behavior towards the pursuit of a new one.

## Acetylcholine, attention, and motivation

The notion that attentional processing can be modulated by motivation seems almost implicit; if one is hungry, they are likely engaging a significant amount of their attentional resources to scanning the environment for signs or cues that direct them to food. Similarly, a student with the goal of receiving high marks is likely to be more engaged and focused on lecture material. A significant amount of research has thus been devoted to understanding this crucial interplay between internal drive states and attentional control, although little consensus exists regarding the precise neurobiological mechanisms that mediate it. A major reason for this lack of consensus stems from the complexity of the constructs of motivation and attention. As reviewed in the preceding text, motivation on its own is a term used to refer to the influence of homeostatic drive states and cognitive representations of desired outcomes on the direction and vigor of ongoing behavior. Similarly, attention may refer to the process of maintaining focus over time, the selection of cues for integration into online decision-making processes (as described above and in Figure 1), or even the filtering out of distracting, task-irrelevant stimuli (e.g., “distractors”). In an influential review, Sarter et al. (2006) conceptualized the specific interactions between attention and motivation as driven by “cognitive incentives”, or internal representations of a goal that drive a subject to maintain attentional control when faced with performance challenges like distractors (Berridge and Robinson, 2003; Sarter et al., 2006). Here, we also focus our discussion on such goal-mediated attentional control mechanisms by first highlighting the major circuitry proposed by Sarter and colleagues. We then extend this model to integrate additional information regarding specific mechanisms revealed by recent studies and propose a shared pathway whereby cognitive and homeostatic drive systems can modulate both cortical cholinergic activity and attentional control.

**FIGURE 2:**
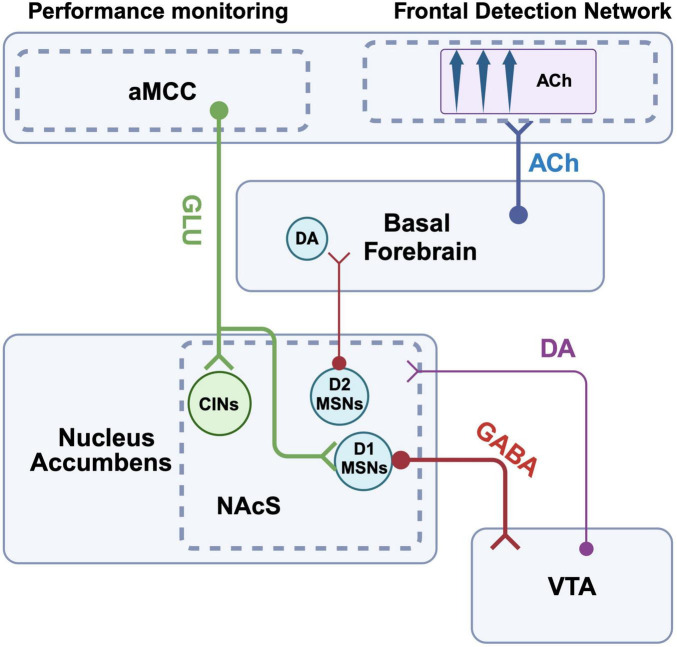
Proposed pathways by which attentional control and frontal cholinergic activity are mediated by cognitive drives. Anterior midcingulate cortex (aMCC) relays information about the loss of task rewards to D1 MSNs in the shell of the NAc. These D1 MSNs in turn project to the VTA and inhibit DA cell bodies. The resulting reduction in DA release also reduces D2 mediated inhibitory drive from the NAcS to the BF, ultimately enhancing ACh release within the FDN and helping to recover detection performance. aMCC projection neurons also target CINs of the NAcS. Recruitment of this descending pathway can increase the activity of CINs, which in turn increase action potential- independent DA release via stimulation of pre-synaptic nAChRs located on DA terminals withing the NAcS. These combined effects on CIN activity and DA release may also help boost the goal-directed motivation to recover task performance. Created with BioRender.com.

To begin, we imagine an individual playing a video game at home. The goal of the game is simple; navigate a race car around a track at high speed. Each player-driver is accompanied by a co-pilot, who signals the direction of upcoming turns by flashing an arrow on a heads-up display. The faster the driver responds to the prompt from the co-pilot, the more efficient their path around the track, and the more likely they are to win the race. In our scenario, the driver is competing against their friends, and we assume that they have a desire to win. The race begins, when suddenly our driver’s sibling enters the room and turns on a television in the background. Curious about the sounds coming from the TV, the driver’s focus begins to drift from the screen. The driver then fails to detect the co-pilot’s subsequent cues, causing them to miss turns and fall behind in the race. As in the scenario above (Figure 1) describing the influence of competing value representations on attentional cue detection, here we also have a competing stimulus that is interfering with the individual’s ability to detect the task events that could be used to guide their ongoing behavior. For the present example, however, we want to highlight situations in which the individual’s ability to detect cues is not being modified by a competing representation of a reward value, or activation of an alternative drive state, *per se*. Rather, a situation in which an abundance of task-irrelevant environmental noise, or distractors, interfere with the driver’s ability to stay on-task. Within this context, we focus on the process whereby the individual recognizes a decline in performance in a game or task that is ongoing, and motivated by their will to win, they volitionally re-double their effort to maintain focus (Figure 2). The model of the brain circuitry that enables the individual to re-engage attentional focus proposed by Sarter et al. begins with this awareness of the impaired performance at the level of the aMCC. The aMCC is an aforementioned part of associative cortex that has long been proposed to be key in the interaction between motivation and attention (e.g., Mesulam, 1981). Animal and human studies provide support that the aMCC is key for the detection of negative events, errors, and more generally calculating cost-benefit ratios during goal-directed task performance (Carter et al., 1998; Walton et al., 2002; Brown and Braver, 2005; Totah et al., 2009; Hillman and Bilkey, 2010; Hayden et al., 2011; Brockett and Roesch, 2021). This information about performance decrements is then relayed from aMCC to the mesolimbic system, specifically the shell of the NAc (NAcS). NAcS is a subregion of the mesolimbic system with a unique pattern of inputs, enriched in markers of cholinergic signaling and neuropeptides, and is generally believed to play distinct roles in reward-guided behaviors relative to other parts of the striatum (e.g., Di Chiara, 2002; Saddoris et al., 2015; Shin et al., 2017; Castro and Bruchas, 2019). Neurons of the NAcS project to the cholinergic BF, and in interaction with local DA signaling have been shown to modulate BF output and ACh release in the PrL of rats (e.g., Moore et al., 1999; Brooks et al., 2007). This pathway represents a critical link between cortical and mesolimbic circuits whereby goal-directed motivation can modify attention. For example, the inclusion of a distracting stimulus decreases performance accuracy in attention tasks, and local infusion of NMDA into the NAcS is sufficient to increase ACh release in the FDN and reverse distracter-induced impairments in performance (Peters et al., 2011). In the model provided by Sarter and colleagues, this capacity of NAcS projections to increase ACh within the FDN, as well as other cortical areas that contribute to attention like posterior parietal cortex, is fundamental in the recovery of attentional performance (Peters et al., 2011). To simplify and summarize the major components of the model by Sarter and colleagues, aMCC error detection neurons recruit NAcS projections to the BF, which in turn amplify FDN (and posterior parietal) ACh release through some unknown mechanism to combat challenges to attentional control (Sarter et al., 2006). Data gathered in the years since this original work supports the translational relevance of this proposed circuitry (e.g., Berry et al., 2017), and has added further granularity to the circuit mechanisms underlying it. Cell-type specific tracing studies indicate that a major target of aMCC input to the NAcS are D1-receptor expressing MSNs (Li et al., 2018). These D1 MSNs of the NAcS project directly to the VTA, and negatively modulate appetitive behaviors typically linked to mesolimbic activity (Bond et al., 2020). These findings imply that output from D1 MSNs in the NAcS hyperpolarizes DA cell bodies of the VTA, an effect that should reduce action potential-dependent DA release and general behavioral impulsivity (Pine et al., 2010; Kim and Lee, 2011; Simon et al., 2013; Weiland et al., 2014). Thus, aMCC input to D1 MSNs of the NAcS may facilitate the recovery of attentional performance in part by first inhibiting VTA output and making behavior more intentional (Flores-Dourojeanni et al., 2021). Improvement in attentional performance, however, should also depend on NAcS modulation of BF cholinergic input to the cortex. NAcS modulation of the BF is not completely understood but it has been shown that local antagonism of D2 receptors in the NAcS is sufficient to increase ACh levels in the FDN (e.g., PrL), likely through actions on local MSNs, but also potentially via D2 receptors located pre-synaptically on glutamatergic inputs (Brooks et al., 2007; Howe et al., 2016). Thus, aMCC input to D1 MSNs, and the inhibition of the VTA, should also reduce D2-mediated activity in the NAcS, and in turn, amplify ACh release in the FDN. Another important target of aMCC projections to the striatum is the local populations of CINs (e.g., Guo et al., 2015). CIN activity in the striatum has generally been linked to the control of goal-directed behavior, including the maintenance of behavioral flexibility and updating behavior based on changing response-feedback contingencies (Ragozzino et al., 2009; Brown et al., 2010; Bradfield et al., 2013). Interestingly, NAcS shows particularly high levels of aceytlcholinesterase, the enzyme that determines the temporal and spatial extent of cholinergic modulation, suggesting this circuit is uniquely tuned to changes in local ACh concentrations (Jongen-Rêlo et al., 1994; Voorn et al., 1994; Shin et al., 2015). The behavioral effects of CINs are due in part to their direct actions on local populations of MSNs (Gritton et al., 2019) which express high levels of mAChRs (Santiago and Potter, 2001), but also through their capacity to modulate terminal DA release via presynaptic nAChRs (Threlfell et al., 2012; Cachope and Cheer, 2014; Kosillo et al., 2016; Brimblecombe et al., 2018). Key to our discussion of the motivated control of attentional performance is the distinction between DA release triggered by changes in bursting activity within DA soma of the midbrain which are typically associated with encoding cue-reward associations (Montague et al., 1996; Schultz et al., 1997; Day et al., 2007; Mohebi et al., 2019), and DA release that is triggered by local presynaptic modulation of DA terminals by nAChRs and correlated with the behavioral pursuit of a goal (e.g., Threlfell et al., 2012; Howe et al., 2013; Cachope and Cheer, 2014; Hamid et al., 2016; Kosillo et al., 2016; Brimblecombe et al., 2018; Mohebi et al., 2019). Recent work has highlighted that CINs exhibit changes in activity during goal pursuit like that described for DA (Mohebi et al., 2023). Combined, we propose that in addition to directly boosting cortical ACh to recover attentional function, and inhibiting action potential-dependent DA release from the VTA, aMCC inputs to the NAcS also recruit local CIN populations, which in turn directly modulate terminal DA release to amplify goal-directed behavior (summarized in Figure 2).

Finally, the discussion above focused explicitly on cognitive, goal-directed recruitment of mesolimbic motivational circuitry to enhance attentional control. However, goal-directed pursuit is not the only context in which motivational drive systems might interact with attentional control mechanisms to optimize performance. Returning to our initial example, consider the context in which an organism is searching for food while hungry. It seems plausible that the brain may also have developed mechanisms to enhance attention in response to such a homeostatic drive, to increase the probability that one detects and can gain access to objects that enhance their likelihood of survival. Within this context, it is important to note that the NAcS also receives abundant input from structures like the lateral hypothalamus, including the population of orexin neurons that are known to encode appetitive drive (e.g., Anand and Brobeck, 1951; Sakurai et al., 1998; Castro and Berridge, 2014a; Castro and Bruchas, 2019; Liu et al., 2020). Lateral hypothalamic orexin neurons also project to the BF, where they can directly modulate cortical ACh release and attentional performance (Arrigoni et al., 2010; Fadel and Burk, 2010; Villano et al., 2017). Thus, we propose that the NAcS may represent a key nucleus in a larger circuitry that allows hypothalamic-homeostatic drives, and frontal-cognitive goal representations, to modulate the BF output to the cortex and enhance attentional control.

### Relevance to disease

Impairments in reward processing and attention are co-morbid across neuropsychiatric and neurodegenerative disorders, as well as drug addiction. We propose that efforts to develop more effective treatments for these symptoms should begin by first defining the specific disruptions in attention and reward that characterize a particular disease state. As an example, we will focus on the impact of a cue that has been paired with the receipt of drugs on attentional control in someone afflicted by addiction. Addiction is associated with increased sensitivity to drug-associated cues (Carter and Tiffany, 1999), and exposure to such drug-associated stimuli would be predicted to simultaneously impair the capacity of task-relevant cues to capture attention and guide behavior, as well as bias motivational control of behavior towards drug seeking over the task at hand (Robinson and Berridge, 1993; Lubman et al., 2000; Paolone et al., 2013). Building off the framework described above, we propose that different circuitries would underlie these two behavioral consequences of exposure to a drug cue. With respect to the capacity of a drug cue to interfere with the detection of task-relevant stimuli, we note that relative to local DA neurons, GABAergic neurons of the VTA seem to be particularly reactive to task-irrelevant stimuli (e.g., Root et al., 2020). We propose that a subset of these neurons project to the BF, are activated by drug cues when encountered during the performance of another goal directed behavior, reduce cue-evoked ACh in the FDN and ultimately interfere with cue detection. It is also worth noting that single-nucleotide polymorphisms in the α5 nAChR is associated with an increased addiction vulnerability for multiple drugs (Saccone et al., 2007; Joslyn et al., 2008). Given the positioning of these subunits across the frontal cortex, including structures within FDN, (Bailey et al., 2010), compounds that selectively boost the function of nAChRs expressing the α5 may enhance the post-synaptic effects of ACh release and simultaneously boost FDN control of the VTA to allow task-relevant cues to maintain control of behavior. With respect to the capacity of these drug cues to re-direct motivated control of behavior, we first draw attention to the importance of CIN modulation of terminal DA release that has been linked to pursuit of rewards (e.g., Hamid et al., 2016; Mohebi et al., 2023). Aside from the aMCC, the OFC is the major prefrontal projection to CINs of the striatum (Schilman et al., 2008; Klug et al., 2018). As described above, the OFC has been linked to a number of reward-related behaviors, and previous studies have shown that repeated exposure to drugs of abuse creates enduring changes in task-related OFC activity (Stalnaker et al., 2006). Interestingly, it appears that different populations of OFC neurons encode drug and non-drug rewards and increases in both the size of the neuronal population as well as the firing rate of those cells seem underlie the decision to seek drugs (Guillem and Ahmed, 2018). Thus, the increase in motivation to seek drugs triggered by drug cues may stem from an abnormally strong input from OFC to CINs and subsequent increase striatal DA release. To combat this drug cue-triggered motivational state, one could imagine that compounds capable of selectively modifying NAcS input to the BF and increases in cortical ACh concentrations could preserve cognitive control of motivation. While targets specific to the BF-projecting population of NAcS neurons are not known, it is worth acknowledging that endogenous opioid signaling mechanisms play an incredibly important role in shaping NAcS contributions to behavior (Castro and Berridge, 2014b; Castro and Bruchas, 2019). As these very mechanisms may also be altered by drugs of abuse, and contribute to the modulation of post-synaptic effects of DA and ACh in the NAcS (e.g., Fiserová et al., 1999; Laurent et al., 2014), future studies should focus on identifying peptide-specific output pathways linking the NAcS to the BF as potential sites of intervention.

## Conclusion

It has long been proposed that the cholinergic system is at the interface between attention and reward. Ours is certainly not the first attempt at accounting for how this ubiquitous neurochemical messenger contributes to each process alone, or in interaction. However, we have attempted to make clear distinctions at both functional and circuit levels, to provide further clarity on the unique processes being mediated by ACh release. Indeed, as mounting evidence continues to suggest, the function of ACh in the brain has for too long been cast as a canonical neuromodulator system; globally rising and falling on relatively slow time scales to support ill-defined global constructs like wakefulness or arousal (Briand et al., 2007; Sarter et al., 2009). This long held idea of ACh function biases interpretations of its relevance to specific functions, which in turn spills over into the design of pharmacological therapies for disorders associated with cholinergic impairments. The clearest example of the impact of this bias is the continued prevalence of acetylcholinesterase inhibitors despite their limited efficacy (Marucci et al., 2021), which likely stems from their non-selective elevation of extra-synaptic concentrations of ACh across the brain. As we have described here, ACh, originating from different cell groups, released in different brain regions, and acting on different receptors, has the capacity to contribute uniquely to a host of different neural computations spanning sensory pleasure to the activation of previously learned action sequences. Similarly, ACh, acting within discrete circuitries that enable reward and attention to interact, can differentially contribute to the control of cue detection and cognitive control of motivation. As always, further understanding of the functions supported by ACh will require further studies. However, true progress in our understanding of its contribution to the neural basis of conscious experience and potential as a target for the treatment of disease necessitates that we as a field acknowledge the functional and regional complexities of this evolutionarily ancient ascending system.
